# Building an interdisciplinary program of cardiovascular research at the Swiss Federal Institute of Technology– the ETHeart story

**DOI:** 10.1016/j.isci.2022.105157

**Published:** 2022-09-27

**Authors:** Andreas P. Kourouklis, Xi Wu, Robin C. Geyer, Vasileios Exarchos, Timo Nazari, Julius Kaemmel, Konstantinos Magkoutas, Marianne Schmid Daners, Miriam Weisskopf, Lucrezia Maini, Cosmin Roman, Jasper Iske, Georgios A. Pappas, Mary Jialu Chen, Caroline Smid, Axel Unbehaun, Alexander Meyer, Maximilian Emmert, Aldo Ferrari, Corina Schuett, Dimos Poulikakos, Edoardo Mazza, Volkmar Falk, Nikola Cesarovic

**Affiliations:** 1 Institute for Mechanical Systems, Department of Mechanical and Process Engineering, ETH Zürich, Zürich, Switzerland; 2 EMPA, Swiss Federal Laboratories for Material Science and Technology, Dübendorf, Switzerland; 3 Laboratory of Thermodynamics in Emerging Technologies, Department of Mechanical and Process Engineering, ETH Zürich, Zürich, Switzerland; 4 Hochschulmedizin Zürich, University of Zürich, Zürich, Switzerland; 5 Department of Computer Science, Institute for Machine Learning, ETH Zürich, Zürich, Switzerland; 6 Product Development Group Zurich, Material and Fabrication, Department of Mechanical and Process Engineering, ETH Zürich, Zürich, Switzerland; 7 Laboratory of Composite Materials and Adaptive Structures, Department of Mechanical and Process Engineering, ETH Zürich, Zürich, Switzerland; 8 Micro- and Nanosystems, Department of Mechanical and Process Engineering, ETH Zürich, Zürich, Switzerland; 9 Department of Cardiothoracic and Vascular Surgery, German Heart Center Berlin, Berlin, Germany; 10 Translational Cardiovascular Technologies, Department of Health Sciences and Technology, ETH Zürich, 8093 Zürich, Switzerland

## Abstract

In this backstory, researchers from Swiss Federal Institute of Technology (ETH Zurich) who initiated an interdisciplinary program to generate innovative solutions for different cardiovascular diseases, such as myocardial infarction, valvular replacement, and movement-based rehabilitation therapy, discuss the benefits and challenges of interdisciplinary research.

**Figure:**
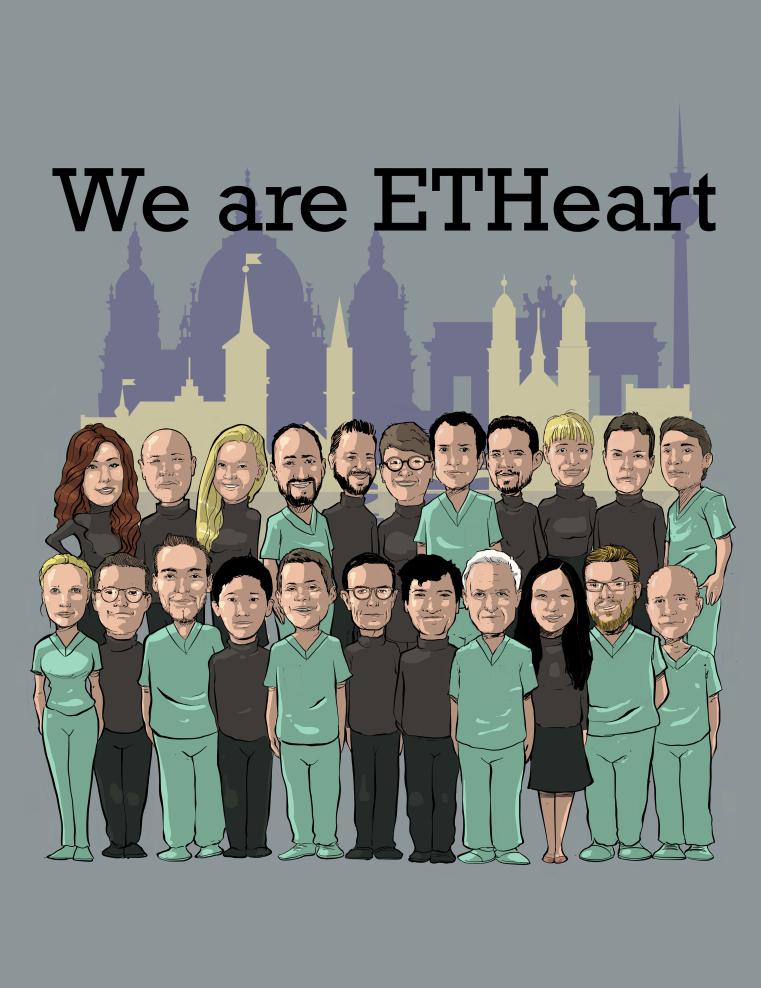
The multidisciplinary team of the ETHeart program with medical professionals from Berlin (green scrubs) and engineers (dark turtlenecks) from Zurich. Illustration created by Payko.

Working directly with medical professionals always reminds me of the fact that I am not working only on the technologies themselves, but also that my solutions could one day be implanted into another human being.

For interdisciplinary collaborations, it is essential to break with hierarchies independent of the academic status of the collaborators.

During patent protection of interdisciplinary medical technologies, it is virtually impossible for one person to cover all the involved aspects.

New strategies to enable the safe reproducibility and comparability of machine learning research on highly sensitive medical information could be of high value for both the medical and the machine learning communities.

## Main text

Cardiovascular disease (CVD) is the leading cause of terminal illness worldwide. Aging population, improper nutrition, and sedentary but stressful lifestyle combined with poor prevention, malignant infections, and high risk-factors (like smoking and obesity) render CVD a leading threat to human health. Life-long treatment, diminished quality of life, repeated hospitalizations, and only short periods of stable health, mark the lives of patients suffering from CVD. In front of this challenge, the Swiss Federal Institute of Technology (ETH Zurich) initiated an interdisciplinary program to generate innovative solutions for different CVDs, such as myocardial infarction, valvular replacement, and movement-based rehabilitation therapy.

Mr. Mayer (not his real name) is a 59-year-old former lightweight boxing champion. His nose is crooked; his eyes are blurry but attentive. As he sits propped up in his hospital bed, he exchanges a couple of sentences with the nurse changing his catheter tube. Yet, for more than that, he runs out of strength. After decades of living life on the edge inside and outside the ring, Mr. Mayer’s heart is failing him. Without a heart transplant, this route has only one destination and the only thing standing between him and the last bell is the small pump implanted within his heart. This machine is what keeps him alive. For now…. Although Mr. Mayer is an imaginary person, our program focuses on improving the lives of patients like him. And what better way to pursue this goal than combining our strengths with cutting-edge science to help patients experience a better quality of life.

### How: Conception and evolution through the years

#### From Zurich Heart to ETHeart

Corina Schuett: Following the principles of complementary collaborations, in 2012 the Zurich Heart Project was launched by “Hochschulmedizin Zurich” (HMZ), a high-level platform to promote large interdisciplinary and interinstitutional projects in Zurich. Under the leadership of Prof. V. Falk (cardiac surgeon), Prof. D. Poulikakos and Prof. E. Mazza (both mechanical engineers), a small group of professors – many of them without a history in cardiac research, but experts in their respective technical fields – began to look intensively into the topic of circulatory support systems. The idea was motivated by the persuasion that there are only a few locations where a large-scale project for the development of such systems can be successful at all; Zurich is ideally positioned for this endeavor. “Hochschulmedizin Zurich” supported the idea right from the beginning with strategic, but also organizational know-how. After several brainstorming meetings, engineers started to look through the glasses of physicians and physicians learned the language of engineers, highlighting the benefits of interdisciplinary collaboration. Nowadays, more than 15 research groups with a large cohort of enthusiastic doctoral students, postdoctoral scientists, and permanent senior scientists work on advanced solutions of cardiovascular technology.

**Figure:**
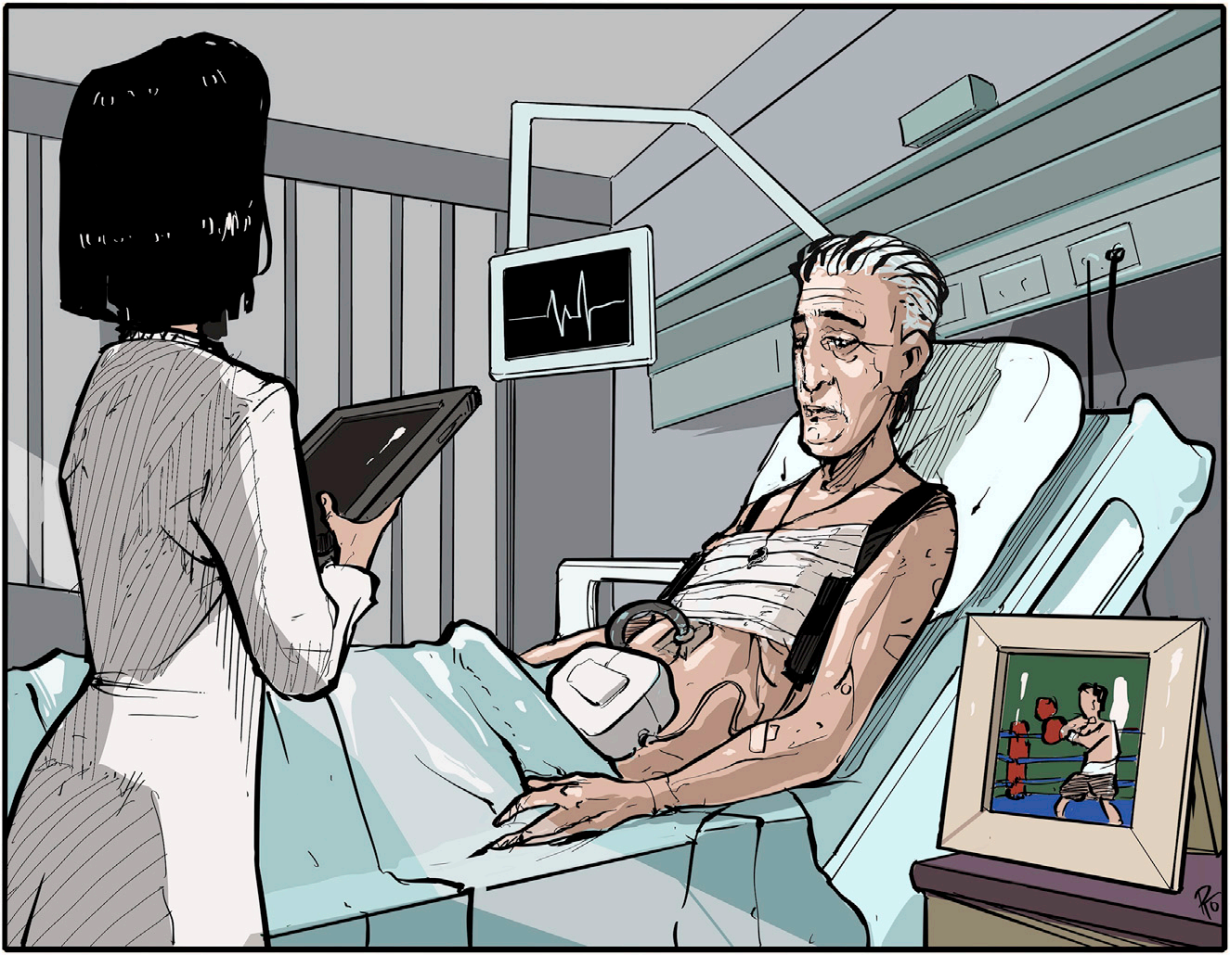
Imaginary, yet typical end-stage heart failure patient relying on an implanted Left Ventricular Assist Device (LVAD) to maintain the necessary blood flow through the body. Illustration created by Payko

### Founders of the program

Who were the players in this project, and how did you bring everyone together? Prof. Falk: The development of medical devices or implants that are to function in the human body is a complex task that is often initially driven by a medical need. Finding a technical solution is demanding from an engineering point of view as strict regulatory boundary conditions set limits to what would be possible from a plain engineering point of view. Developing technical solutions in the framework of a biological system such as the human body requires a constant collaborative effort between physicians and engineers. The will to understand and communicate each other in a common language can only develop over time and mandates that frequent interaction during the development process take place. This can only happen if there is also excitement for a joint project. The Zurich Heart was such a project because it motivated scientists from the ETH to combine their skills to develop an alternative to the current generation of ventricular assist devices, which have several limitations. After visiting patients in the ICU, and with a good understanding of the technical constraints and limitations of the available systems, a group of enthusiastic ETH scientists began to tackle some of the most compelling challenges including but not limited to new sensors and control algorithms, transcutaneous energy transfer systems, optimization of pump design, and the development of biological interfaces. New materials and technologies were brought into the field, but also wholly new concepts such as a soft pump design were explored.

#### How did the decision of branching out of your fields come about?

Prof. Falk: Before I came to Zurich, I had also experienced very fruitful collaborations with ingenious engineers from the Technical University in Karlsruhe, the French Institut National de Recherche en Informatique et en Automatique, the Deutsche Luft-und Raumfahrtzentrum and had founded the Innovation Center for Computer Assisted Surgery (ICCAS) in Leipzig. The focus then was mainly on robotic systems and image-guided planning of cardiovascular procedures. It was clear that none of the developments would have ever been possible without crossing the traditional boundaries of the medical school environment. What would have been more natural than tapping into the huge resources at ETH which literally offers expertise in any possible field that is relevant to advance medicine?

#### What implications did it have on your careers? (Recognition, impact, teaching, funding, etc?)

Prof. Falk: The collaborations were well recognized although some basic research went almost unnoticed in the medical arena. This is largely because the IEEE Journal and other more technical journals are unknown to most physicians. It is usually later in the translational process when the medical field starts to show interest in new technology. In terms of teaching, one had to adapt to a different audience that also thinks differently about a medical problem. This understanding helps to develop a common language or “ontology” which largely facilitates the interaction.

#### What did you learn about interdisciplinary research from the project and what tips would you give to anyone considering undertaking such work? Are there any other challenges you encountered that are not discussed here?

Prof. Falk: It is unfortunate that many of the projects strictly follow the classic “post-Doc” cycle. This cycle usually leads to a patent and a decent paper as well as a prototype and sometimes even early large animal experiments to prove that the principle works not only in silico but *in vivo* (one of the major steps in translation). Often, once this goal has been achieved, the career path of the involved scientist progresses outside the ETH and the project comes to a sudden end. But that a technical solution is found does not mean the task is finished. Before an idea becomes a medical product typically multiple iterations are required. Those may look less inspiring and no longer fall in the scope of pure science. In addition, proper documentation which is essential for any regulatory process ahead is often missing. This gap needs to be filled and I believe that this discussion must be led by the Schulleitung.

Prof. Mazza and Prof. Poulikakos: Zurich Heart and ETHeart started as bottom-up initiatives, based on spontaneous interactions between research groups. Zurich Heart began with presentations by Prof. Falk, indicating the main open problems toward the use of ventricular assist devices (VADs) as destination therapy for heart failure patients. Several sessions followed in which all interested research groups discussed new ideas and suggested new solutions for systems of cardiovascular support. A collegial atmosphere fueled prolific discussions over non-conventional ideas and specific solutions for improving existing devices. These open discussions and the inclusive spirit were key to forming a large and engaged community, contributing to a common endeavor. Interestingly, there was no promise of financial support at the start of Zurich Heart. We are convinced that all PIs were genuinely motivated by the unique opportunity to work on high impact applications associated with CVD.

#### How did the decision of branching off from your fields come about?

Prof. Mazza: I believe that transdisciplinary research represents the main challenge for scientists and engineers of our generation. We experience a sort of globalization among scientific disciplines, which creates completely new opportunities for both, technological innovation and fundamental investigations. This trend is stronger in academia, but it is increasingly important also in industry. For this reason, it is an important part of our mission to prepare the new generation of engineers and scientists to tackle transdisciplinary challenges, to develop a language that allows interdisciplinary communication but without affecting scientific depth.

#### What did you learn about interdisciplinary research from the project and what tips would you give to anyone considering undertaking such work?

Prof. Mazza: In my view, the most important component of interdisciplinary research is the mutual appreciation of the relevance of each discipline. It needs some efforts to drop a natural defensive view and to understand and appreciate the top-level competences required to manage the complexity of the “other” field. Institutions can provide incentives, but successful interdisciplinary projects are based on bottom-up initiatives, driven by the enthusiasm for a common goal toward which each discipline provides essential input.

#### Are there any other challenges you encountered that are not discussed here?

Prof. Mazza: Fruitful interaction requires time, and we are all lacking time. Despite the good will of each participant, on several occasions I had the impression that the output could be stronger if we had more time to understand each other’s viewpoint. Although this is probably an unavoidable limitation for the PIs, it is important to motivate younger researchers to invest sufficient time with their colleagues in the interdisciplinary community and learn as much as possible from each other. The ETHeart Joint Scientific Colloquium represents a great opportunity for such exchanges.

### Life within an interdisciplinary project

#### Did you see any benefits and challenges from working as part of an interdisciplinary team of engineers and clinicians? What steps did you take to enhance collaboration?

Vasileios Exarchos: As a young physician with an internal medicine background, in the German Heart Center Berlin I jumped at the opportunity to tend to patients with permanent left VAD (LVAD). Especially, the complex long term care of these patients, that include common severe complications such as thrombosis of the pump, were of particular interest during my clinical practice. However, day after day, enthusiasm turned into contemplation or even frustration. The inner metallic surface of LVADs has reduced hemocompatibility leading to blood activation and thrombus formation with devastating thromboembolic complications without any satisfactory solutions. The idea to tackle this major clinical problem with research came to me when I heard about the research performed within the Zurich Heart Project in 2018. I started working together with experts in the field of mechanical engineering and mechanobiology within the context of the Zurich Heart Project. We are trying to investigate the following hypothesis: What if we cover the VAD luminal inner surface with a layer of living endothelium to increase the grade of hemocompatibility? This is how my journey in the uncharted waters of mechanobiology began. Now, only a few meters away from the clinical department where I work, we perform live cell imaging of endothelial cells, direct isolated from our cardiovascular patients as a regular instrument to understand the development of living endothelium on foreign surfaces. We analyze 3-dimensional data, using fluorescence microscopy as well as specific computational codes, to investigate the interplay between nanosized endothelial cells particles and various micro structured surfaces designed at ETH Zurich to facilitate endothelialization. How will this journey end? I do not know. However, the lesson I am not going to forget is our way to confront scientific problems from the perspective of everyday clinical life.

#### What are the benefits and challenges of working with clinicians?

Xi Wu: Working directly with the medical professional always reminds me of working not only on the technologies themselves, but also on the fact that my solutions could one day be implanted into another human being. This fills me with pride but also certain fear in face of such responsibility. There are many practical, real-life limitations that could introduce significant risk into the implementation of a promising technology in a stressful environment such as the cardiovascular operating theater. Hence it is very important to talk to clinicians frequently for input. To achieve good communication, I found that using graphical tools such as 2D illustration or 3D renderings was very useful to convey my concepts and solutions.

#### What steps did you take to enhance collaboration with engineers (clinician’s response)?

Vasileios Exarchos and Timo Nazari: We visited the laboratories of our partners at ETH Zurich quite frequently, although in-person visits were quite difficult during the past 2 years. Initially concepted as a collaboration where we provide human tissues and cell lines for device testing, it quickly developed into deep conversations about our viewpoints on the science of our projects and usually sparked new research ideas. As one might say, we came with suitcases full of biological materials (and ideas) and never left empty-handed. To allow such a collaborative environment, junior team members such as Xi and Vasileios need to feel an environment where it is okay to ask questions and even challenge ideas no matter from whom they come. Even today, clinicians are usually embedded in a system of hierarchy (especially surgeons) where this kind of communication is not encouraged. For interdisciplinary collaborations, it is essential to break with these hierarchies independent of academic status of the collaborators. Each of our engineering students and medical/biomedical students is usually mentored by a more senior scientist in that respective field. However, the responsibilities of mentoring are shared with low thresholds for communication within the team. It is also important to find a common language for conveying information between the worlds of engineering and medicine. That also includes creating a common patient centric goal. It is therefore as important to introduce engineers to every day clinical life as it is now very common for medical doctors and students to at least spend some time in the engineering laboratory. We allow also non-medical scientists to observe surgeries and partake in clinical rounds in the wards and intensive care units (ICUs) to demonstrate processes and spark motivation.

#### What benefits and challenges did you encounter by collaborating with people from different backgrounds and expertise while organizing preclinical trials?

Andreas Kourouklis and Julius Kaemmel: Drivelines connect LVADs with the battery and control unit located outside their body. As it breaches through the skin, the driveline causes a permanent wound which is vulnerable to bacterial contamination and infection, which can quickly spread along the driveline to the heart of the patient. For this reason, we pursued the design of new driveline systems with the aim to inhibit bacterial infection in well-designed preclinical studies. Andreas Kourouklis: We found ourselves puzzled as to how to perform the surgical implantation of the *in vivo* trial in a clinical-like fashion.

Julius Kaemmel: From a clinical standpoint, we had to come up with a new surgical scheme that accounts for both the technical features of the implant and the principles of approved clinical protocols. Nikola Cesarovic: We also came across some unique challenges while designing this study. Our initial plans could not have predicted the limited amount of study items and lengthy delays in the production process caused by the pandemic. Moreover, in contrast to the recovering LVAD patients, the study animals will not quietly lie in bed. Hence, our finally adapted solutions took some out-of-the-box measures to ensure wound protection and stress-free animal conditions.

Konstantinos Magkoutas: I echo what has been mentioned above and I will add some experiences from my own project. Following successful *in vitro* and in-silico testing of a hall-based sensor for monitoring major hemodynamic parameters, the time had come to perform the first *in vivo* trials. Initially, the decision of a testing scheme was quite challenging because our sensor device needed to be implanted on a major artery before assessing a wide range of hemodynamic parameters. However, partnering with Nikola and Miriam’s team of laboratory animal experts made the decision on study design and animal model rather straightforward. I learned to create primary and secondary endpoints for the trial and have a clear prioritization of the data that we really need to obtain.

Nikola Cesarovic: Owing to the specific experimental setting and the fact that the surgical team had to be kept to a minimum because of pandemic rules, Konstantinos was always with us in the operating room to provide input, clarify questions, and tackle technical issues on the spot.

Miriam Weisskopf: This experience just confirmed that with mutual respect collaborators with different but complementary expertise can quickly and satisfactorily accomplish even the most challenging projects even under unprecedented conditions such as the COVID-19 pandemic.

#### What benefits and challenges did you experience by collaborating with people from different backgrounds and expertise, while protecting your technology through patent application?

Lucrezia Maini and Cosmin Roman: Our experience with the medical team has been very positive and proficient. From the very first day of my PhD, we have been in contact with Nikola, who has been very open to discuss and illustrate the critical aspects related to the medical challenges of the project. It has certainly been a team effort so far, from everybody involved in the project! The most remarkable experience we had was the drafting of our first patent. It was the first time for us, and thus quite fascinating. Since then, we conceive patenting not only as a means to protect an invention but as a source of innovation as well. Patenting an invention requires a deep knowledge of the state-of-the-art, both from an industrial and academic point of view. During patent protection of interdisciplinary medical technologies, it is virtually impossible for one person to cover all the involved aspects. Hence, the interaction with clinicians and translational scientists was of main importance not only to establish the medical specifications of our designed sensor but also to compare its features with the state-of-the-art.

In addition, during the patenting process we collaborated with legal and technology transfer experts, whose understanding of the invention is crucial for securing intellectual property (IP) protection. Together with our colleagues, we pondered for hours over what specific word or phrase should be used to effectively describe the features of the invention. Nikola Cesarovic: We quickly realized that the same things could have completely different meanings in engineering, medical, or legal terms and that none of us can rely on our familial terminology to adequately describe the novelty of, e.g., the sensor. Lucrezia: As a first step in this process, I performed an assisted patent search together with experts from the Swiss Federal Institute of Intellectual Property in Bern (yes, the place where Einstein worked). This is the moment that an invention is evaluated relative to its novelty, application range, and inventiveness point of view, which are all necessary to determine patentability. During this process, the most representative keywords related to the invention are searched at ESPACENET, before a concluding report is issued by the patent officer. I think this is a critical task of the process because the correct selection of keywords is important and may influence the patentability of an invention. After this step, we started working closer with our patent attorney and medical partners to construct the claims of the invention. Interestingly, the most challenging aspect in this final stage was the communication with the patent attorney who needed to decode the invention quickly and in depth before they move on to define and protect its IP.

#### What benefits and challenges did you encounter by collaborating with people from different backgrounds and expertise while applying for research funding?

Jasper Iske: The combination of different scientific backgrounds offers diverse scientific funding opportunities. There is more to this play on words than just a proverbial phrase. This is because diverse scientific funding opportunities not only refer to the frequency and range of application possibilities. At the same time, an interdisciplinary project introduces a new perspective into a field of research, which in turn can provide solutions to a pressing clinical challenge. Recently, we developed an interdisciplinary strategy to characterize pathological mechanisms by developing diagnostic, therapeutic, and prognostic biomarkers in collaboration with veterinary experts from ETH Zurich and the German Heart Center Berlin. This collaboration enabled us to pursue funding from three synergistic fields of this project, including biophysics, preclinical studies, and basic immunology.

Nikola Cesarovic: However, not all is rosy. Multidisciplinary projects are increasingly complex with the risk of becoming overwhelming. Such projects are suited only for the largest benefactors. Hence, it is significant to conceive a project scheme that is suitable for a wide range of funding opportunities. Ideally, the creation of several sub-projects can reduce the overall complexity and shorten the timelines for obtaining the associated results. Later, these projects can be freely combined to pursue additional funding. Furthermore, the drafting of grant proposals is an energy- and time-consuming process that regularly remains unrewarded. To avoid terminal frustration, we have been viewing the composition of funding proposals as an excellent opportunity to acquire preliminary results and test various hypotheses.

#### How did you receive teaching and learning activities by being part of an interdisciplinary audience? What is the ideal strategy for effective cross-disciplinary training?

Nikola Cesarovic: In my previous experience as the head of experimental surgery unit, I had the occasional pleasure of working with engineers so profoundly proficient in the medical aspect of their project that I would be hard pressed to discern between them and a trained medical doctor. However, such expertise is rare, and it requires years if not decades to develop. Personally, I believe it is more readily observable in medical industry then in academia. In our program, we try to foster interdisciplinary collaboration by providing students with the formal tools of the trade. The Joint Scientific Colloquium of ETHeart provides engineers with the fundamental knowledge about heart medicine while at the same time creating a platform for idea exchange and feedback.

Wu Xi: A very important step is to learn about another field that is fundamentally different than the one I was trained in. Several concepts of the biomedical sciences are not intuitive for me as an engineer. However, the more I learn, the more I appreciate how complicated and intricate the human body is. In this process, I read as much as possible, take relevant courses, pursue the advice of experts as and learn through practice. I personally think that by not being afraid to ask is the first step to obtain training into a new scientific field.

Georgios Pappas, Mary Chen, and Caroline Smid: A great challenge in our collaboration is the communication of concepts in an interdisciplinary audience, i.e., engineers need to understand physicians and vice versa. To be more precise, engineers often need to communicate problems and solutions to medical doctors or translational scientists and strongly rely on direct feedback before making significant progress. Thus, we need to communicate the true essence of our findings without diluting critical information. To be effective, such communication should be built on some (at least basic) common knowledge among partnering scientific fields. Eventually, the developed learning process is progressive, area focused, and problem based. We acquire and communicate knowledge by real life examples sourced from our project. Interdisciplinary learning is a work-intensive process, yet when we all communicate at a high level we can pioneer solutions to problems that are yet to be solved.

Axel Unbehaun: By combining patient safety and therapeutic efficacy, clinicians tend to follow the moto ‘use what is proven to work’. Consequently, we are frequently hesitant to new therapeutic developments, especially if they largely differ from the current state of the art. I would say that engineers offer the stimulus to think out-of-the-box which is pivotal to exploring new therapeutic protocols. For medical doctors, a great part of our learning experience is simply based on the perspective of our collaborating engineer. Through such interactions, we can also find ourselves in front of technologies which are just waiting for their use in medical applications. Hence, I would say that my interaction with engineers is not only a learning experience but also a growing journey.

#### When publishing this or any interdisciplinary paper, how do you decide which community/venue to target? What are the challenges during publication of this or any such research? What initiative (by publishers, funders, etc.) would make communicating interdisciplinary research easier/more effective?

Robin C. Geyer and Alexander Meyer: The overarching goal of our study is to identify data patterns associated with critical events in ICU patients and subsequently to investigate how machine learning could be leveraged to detect such patterns early and reliably. On the one hand, the data collection platform designed for the study and the identification of specific data patterns can be of high interest for the medical community and subsequently for its publishers. On the other hand, the multimodal, high-resolution dataset collected during the study will enable the data-driven development of new machine learning methods, which can be of high interest to the machine learning community and its publishers.

Reproducibility plays an important role in machine learning research. Ideally, when an article is published, the relevant dataset, model and codebase are also published. However, in medical applications special caution is to be taken to protect patient information. Ensuring differential privacy when publishing data and trained model can be extremely challenging for researchers. We believe that new strategies to enable the safe reproducibility and comparability of machine learning research on highly sensitive medical information could be of high value for both the medical and machine learning communities.

